# Scope and challenges of machine learning-based diagnosis and prognosis in clinical dentistry: A literature review

**Published:** 2021-07-30

**Authors:** Lilian Toledo Reyes, Jessica Klöckner Knorst, Fernanda Ruffo Ortiz, Thiago Machado Ardenghi

**Affiliations:** Department of Stomatology, School of Dentistry, Federal University of Santa Maria, Santa Maria, Brazil

**Keywords:** artificial intelligence, machine learning, dentistry, oral health, diagnosis, prognosis

## Abstract

**Background:**

Machine learning (ML) has emerged as a branch of artificial intelligence dealing with the analysis of large amounts of data. The applications of ML algorithms have also expanded to health care, including dentistry. Recent advances in this field point to future improvements in diagnostic techniques and the prognosis of various diseases of the teeth and other maxillofacial structures.

**Aim:**

The aim of this literature review is to describe the basis for ML being applied to different dental sub-fields in recent years, to identify typical algorithms used in the studies, and to summarize the scope and challenges of using these techniques in dental clinical practice.

**Relevance for Patients:**

The proficiency of emerging technologies that have begun to show encouraging results in the diagnosis and prognosis of oral diseases can improve the precision in the selection of treatment for patients. It is necessary to understand the challenges associated with using these tools to effectively use them in dental services and ensure a higher quality of care for patients.

## 1. Introduction

Artificial intelligence (AI) can be defined as the non-biological ability of a machine trying to imitate human intelligence to accomplish complex tasks, such as problem-solving, object and word recognition, and decision-making [1-4]. The first reports of its applicability in Medicine appeared in the early 1970s, with the advent of some experimental computer systems [5,6] and new surgical research by Gunn, who explored the possibility of diagnosing acute abdominal pain with computational data analysis [7].

The major advances in the use of AI techniques in Medicine have been limited to the prognosis and diagnosis of diseases or health events, thereby enabling clinical decision-making through the application of machine learning (ML) algorithms that use supervised learning [8]. Accordingly, several examples support the promising findings that have resulted from the application of these technologies, further encouraging their use [9-15].

The use of ML has also been expanded to other domains of healthcare, including dentistry. According to the PubMed repository records, reports describing the favorable results of the application of these techniques in various clinical disciplines in dentistry began to appear in the early 1990s. The scientific literature on the subject highlights orthodontics as one of the specialties where the use of ML has brought palpable benefits, especially through the extraction of characteristics from radiographic images [16-22]. Many studies using data mining have yielded reliable results for differential diagnosis of a wide range of oral diseases [23,24]. These reports indicate high accuracy in diagnosis, allowing the standardization of procedures and time optimization in the analysis of large databases. In addition, the previous studies report advances in Cariology, both in the generation of predictive risk models and in the estimation of useful patterns in the diagnosis of dental caries [25-32]. Thus, the recent application of ML in these fields may lead to future improvements in diagnostic techniques and in the prognosis of various diseases of the teeth and other maxillofacial structures [6].

However, there are few records that summarize the primary applications of ML in the wide spectrum of dental sub-fields and discuss the key factors guiding the application of these technologies in dental practice. Although the subject has gained importance in recent debates, most of the previous reviews focus on areas such as dental imaging and address specific algorithms. This is comprehensible if we take into account that the use of ML may not be the best solution for every problem. Even so, in recent times, there are several reports that bring the use of ML to other dental disciplines. Therefore, it is crucial to identify the impact of these techniques in the broad field of dentistry. Further, the challenges associated with the clinical application of these tools, such as the selection of dental datasets and reference standards, need extensive debate, and discussion [4,6,33-35]. A comprehensive analysis of the current use of these techniques to support the decision-making of professionals would be of great interest to improve clinical judgment, reduce errors in diagnosis, and lower treatment costs in the dental office environment.

In this review, we describe the basis for ML being applied to dentistry and summarize the primary ML-based approaches in different dental sub-fields in recent times. We also analyze the typical algorithms used in studies that apply ML methodologies. Finally, we discuss some key factors that may guide the practical implementation of these technologies based on the current trends in this field.

## 2. Fundaments of ML

### 2.1. ML

Recent years have seen rapid growth in data recording efforts due to the ability of computer systems to store and share large volumes of information. This explosion of information has often been called “big data.” Therefore, it has become necessary to develop new procedures that combine statistical (mathematical) and computational patterns in the analysis and critical interpretation of datasets, to obtain valid criteria and make effective predictions [36]. In this context, ML emerges as a branch of AI that involves the analysis of large amounts of data [2]. ML algorithms learn from the previous examples, performing useful inferences to assist decision-making [37,38]. In the ML environment, the goals are focused on the recognition of significant data patterns within datasets as well as in proposing models that best explain the data [39].

### 2.2. ML paradigms

At present, the advances in these technologies allow for distinguishing between various types of learning methods and algorithms for data pattern recognition. Supervised, unsupervised, and reinforcement learning paradigms have been widely accepted for this purpose [39-41].

In the domain of supervised learning, ML operates through mapping functions between the available input and output variables [39]. It contributes to the enrichment of the analysis, bringing them closer to true or established criteria by involving labeled variables. This offers an advantage for its implementation in medical practice, replacing or complementing expert opinions in solving various problems. Supervised learning is recognized as the most widely used and promising approach in this field. These techniques had been generally used in dentistry for the prediction of some events or diseases [25,42], as well as in the diagnosis and classification of specific dental and oral-maxillofacial conditions [43-45].

In contrast, unsupervised learning approaches involve algorithms that are executed only from input data [39]. Here, the algorithm is trained for finding similarities, allowing for non-linear and interactive combinations of various predictors, identifying patterns in the data, and achieving reliable results in the analyses performed. Unlabeled dental patient databases allow for the recognition of labels like those related to certain patterns of bone loss associated with periodontal disease. This can create clusters that allow further analysis. The techniques described above can also be combined in a semi-supervised approach [46].

Finally, reinforcement learning can be represented as an extension of dynamic programming techniques, in which the underlying model is arrived at through complex mechanisms of positive reward and punishments [47]. In dental clinical practice, the high precision of algorithms using reinforcement learning has become evident in applications involving image processing [38,48-51].

#### 2.2.1. Clinical Applications of ML Algorithms in Dental Practice

We conducted a review of original studies that reported applications of ML algorithms in different fields of clinical dental practice published during the past 10 years. The electronic literature search included publications from the PubMed/Medline database up to March 2021, as well as some references cited in the reviewed papers. The search strategy employed a combination of controlled vocabulary and keywords: “artificial intelligence”[MeSH Terms] OR “machine learning”[MeSH Terms] OR “supervised machine learning”[MeSH Terms] OR “unsupervised machine learning”[MeSH Terms] OR “deep learning”[MeSH Terms] AND “prognosis”[MeSH Terms] OR “diagnosis”[MeSH Terms] OR “treatment”[MeSH Terms] AND “dentistry”[MeSH Terms] OR “oral health”[MeSH Terms] OR “teeth”[MeSH Terms] OR “orthodontics”[MeSH Terms] OR “periodontal diseases”[MeSH Terms] OR “operative dentistry”[MeSH Terms] OR “oral surgery”[MeSH Terms] OR “prosthodontics”[MeSH Terms] OR “endodontics”[MeSH Terms] OR “implants and prostheses”[MeSH Terms] OR “ dental implants”[MeSH Terms] OR “pediatric dentistry”[MeSH Terms] OR “dental radiography”[MeSH Terms] OR “radiology”[MeSH Terms] OR “forensic dentistry”[MeSH Terms] OR “oral medicine”[MeSH Terms] OR “maxillofacial surgery”[MeSH Terms]. Review papers, case series, case reports, editorials, letters, comments, conference abstracts, and papers limited to describing educational methodologies or the use of robotic devices were excluded from the search. A total of 542 titles were initially identified. After analyzing the abstracts of the studies that met the eligibility criteria, 62 papers were included in this review. In this review, we provide detailed comments on these findings and discuss some interesting aspects of the main ML algorithms used in these reports.

### 3.1. Applications in orthodontics(Table 1)

The vast majority of ML applications in dental clinical practice appear to be linked to diagnostic abilities. In this sense, ML algorithms have allowed us to optimize and improve the use of available data in Orthodontics, with substantial contributions to the diagnosis of dental maxillofacial anomalies for the assessment of treatment needs through the use of training datasets containing radiographic images [16,17,19,20,22,50,52-57].

**Table 1:** Main features of surveyed studies using machine learning algorithms in orthodontics

Dentistry field	Application	Data set	Machine learning algorithms	Performance
Orthodontics	AI system for automatic location of cephalometric landmarks Leonardi *et al*. [16] (2010)	40 lateral cephalometric radiographs, from subjects; age range 9 -15 years; mean age13.9 years	Cellular NNa	Differences between the two methods P<0.05
	
	Predict the need for extractions in orthodontic treatment with the use of an expert system in patients between 11 and 15 years of age Xie *et al*. [17] (2010)	200 patients; age range 11-15 years	ANNs^b^	Accuracy: 80%
	
	Diagnosis of dental deformities in cephalometry images through extraction of landmarks for craniofacial features on cephalometric radiography Banumathi *et al*. [53] (2011)	100 lateral cephalometric images	Gini SVM^c^, naïve Bayes	Accuracy: proposed Gini SVM, 98%; existing Gini SVM, 92%; naïve Bayes, 67%
	
	Predict the sizes of unerupted canines and premolars during the mixed dentition period Moghimi *et al*. [18] (2012)	106 untreated subjects (52 girls, 54 boys); age range 13-15 years	Hybrid GA - ANNs^d^	ICC^e^: 0.697, mandible; 0.742, maxilla
	
	Develop an expert system to improve the diagnosis and the decision to extract teeth for orthodontic treatment Jung and Kim [19] (2016)	Lateral cephalograms of 156 patients	ANNs	ICC: 0.97 to 0.99; success rates: diagnosis of extraction/ nonextraction, 93% ; diagnosis of the extraction patterns in total, 84%
	
	Assessing orthodontic treatment need and outcome from the lay perspective Wang *et al*. [20] (2016)	Visual scan paths of 88 laypersons	SVM^f^	Accuracy: treatment need, 97.2%; treatment outcome, 93.4%
	
	Automatic teeth detection and numbering Chen *et al*. [54] (2019)	1250 periapical radiographs	CNNs^g^	Precisions and recalls exceed 90%; IoU^h^,, 91%
	
	Decide stages of growth and development of use in Orthodontics by means of measurements of the vertebrae Kök *et al*. [55] (2019)	Cephalometric radiographs, from 300 individuals; age range 8-17years	k-NN^i^, naïve Bayes, DTj, ANNs, SVM, Random forest	Accuracy: k-NN, CVSk 5 (60.9%), CVS 6 (78.7%); naïve Bayes, CVS 1 (992.1); DT, CVS 1 (97.1%), CVS 2 (90.5%); ANNs, CVS 1 (93%), CVS 2 (89.7%), CVS 6 (78%), CVS 3 (68.8%), CVS 4 (55.6%), CVS 5 (47.4%); SVM, CVS 3 (73.2%), CVS 4 (58.5%); Random forest, CVS 5 (36.8%)
	
	Describe the impact of orthognathic treatment on facial attractiveness and age appearance Patcas *et al*. [21] (2019)	2164 pre and post treatment photographs of 146 orthognathic patients	CNNs	Similarly and beneficial effect on attractiveness in 74.7% (MD^l^: 1.22 (95% CI^m^: 0.81;1.63); *P*<0.001)
	
	Assessment of facial attractiveness of patients who have undergone treatment for clefts using an automated system Patcas *et al*. [61] (2019)	Frontal and profile images of 20 treated left-sided cleft patients (10 males; mean age: 20.5 years) and 10 controls (5 males; mean age: 22.1 years)	CNNs	Mean score: 4.75 ±1.27; all *P*≥0.19
	
	Automatic analysis of panoramic radiographs for teeth detection and numbering Tuzoff *et al.* [56] (2019)	1352 panoramic radiographs of adults	CNNs	Teeth detection: Se^n^, 0.994; precision, 0.994 Teeth numbering: Se, 0.980; Sp^o^, 0.999
	
	Evaluation of the variation of the maxillary structure in unilateral canine impaction Chen *et al*. [52] (2020)	107 CBCT^p^ images	Random forest	ICC: 0.994 (auto-segmentation); 0.999 (auto-landmark)
	
	Orthodontic diagnosis using an automated cephalometric X-ray analysis Kunz *et al*. [22] (2020)	1792 cephalometric radiographs	CNNs	ICC: > 0.864, p< 0.001
	
	Automated measurement of mandibular molar angulations on panoramic to predict the potential for eruption of third molars Vranckx *et al*. [50] (2020)	838 panoramic radiographs	CNNs	mean IoU: 90%; accuracy: 79.7% (–2.5°; 2.5°) and 98.1% (–5°; 5°)
	
	Classification of impacted maxillary supernumerary teeth Kuwada *et al*. [57] (2020)	550 panoramic radiographs (275 patients with at least 1 impacted maxillary supernumerary teeth (ISTs) and 275 patients without ISTs in the maxillary incisor region	CNNs	AUCq (Model C, DetectNet): 0.93 (testing data 1); 0.96 (testing data 2)

^a^Cellular NN: Cellular neural networks;

b ANNs: Artificial neural networks;

c Gini SVM: Gini support vector machine;

d Hybrid GA-ANN: Hybrid genetic algorithm and artificial neural network;

e ICC: Intraclass correlation coefficient;

f SVM: Support vector machine;

g CNNs: Convolutional neural networks;

h IoU: Intersection-over-union;

i k-NN: k-nearest neighbors; ^j^DT: Decision tree; ^k^CVS: Cervical vertebrae stages;

l MD: Mean absolute difference;

m CI: Confidence interval; Se

n :Sensibility; Sp

o :Specificity;

p CBCT: Cone beam computed tomography,^q^AUC: Area under receiver operating.

A previous study in this sub-field successfully extracted the landmarks for craniofacial features and analyzed cephalometric variables to provide accurate diagnoses of dental deformities [53]. Standard procedures for cephalometric analyses are liable to multiple errors resulting from frequent deviations between the locations of various observers. AI techniques contribute to improving these procedures. In this proposal for automated dental deformity diagnosis methods, support vector machine (SVM) algorithms showed the best performance (98% accuracy) in comparison to the accuracy of other classifiers. SVM-based models have gained popularity in the ML community. These classifiers are the most effective means of separating data in multidimensional space [58,59]. It is possible to use this method to reduce the requirement for the number of images and increase the speed of the analysis when compared to the performance of the dentist. SVM algorithms have been described as appropriate for learning tasks in which the number of characteristics is large. Moreover, the complexity of SVM is not altered by the number of functions found in the training set, which is a desirable property for its practical applications [60].

Several studies point to the success of the application of different types of neural networks algorithms in the segmentation, automatic detection, analysis, and extraction of image features, to enable more effective diagnosis in Orthodontics [16-19,21,22,50,54-57,61]. A recent study that used convolutional neural networks (CNNs) algorithms with a large dataset achieved high-quality training, generating a high-precision model for the analysis of cephalometric data. The model corresponded well with the criteria chosen by experienced human examiners, thus meeting the gold standard at a faster rate [22]. The use of CNNs algorithms has been widely disseminated in dentistry and it is recognized that these algorithms can create significant improvements in the quality of images by reducing dispersion and artifacts [35,41]. These deep learning algorithms are a priority in complex tasks in which there is a large amount of unstructured data, as would be the case with the classification of dental images, where their effectiveness has already been established. These classifiers have the ability to recognize hidden relationships between interdependent variables and estimate decision rules. They are preferred when precision is prioritized. However, classical algorithms are more useful for simple tasks in which there is no unstructured data and the interpretability of results is of higher priority [22,35,41].

Recent research has encouraged the use of neural networks for prediction tasks [18]. Hybrid genetic algorithms and artificial neural networks (ANNs) have been implemented in order to establish a prognosis of the size of non-erupted canines and premolars during the period of mixed dentition. The impacts of orthognathic treatment on facial attractiveness and age appearance for common patients and patients who have undergone cleft treatment have also been explored by applying CNNs [21,61]. One particular study does not recommend the application of CNNs algorithms in some cases [61]. However, the report describes possible limitations related to the characteristics of the study population and the specific scores used to measure the perception of physical attractiveness. These limitations may have contributed to the negative outcome of using CNNs.

It is necessary to emphasize that to achieve high clinical precision with the use of neural network algorithms, it is advisable to increase the training data set, obtain better estimates of the weights of the connections, and increase the classification and predictive power. Furthermore, hybrid dental data collected on a large scale from multiple healthcare institutions and public registries could be useful during the training phase. Interested researchers should collaborate and share their data collections to bring new advances in this field [62].

### 3.2. Applications in periodontics (Table 2)

In the field of periodontics, ML algorithms have demonstrated a good performance in working with molecular profile data, immunological parameters, bacterial profiles data, or clinical and radiographic variables of affected patients [42,63-67]. Identification of bone levels, identification of bacteria in subgingival fluid samples, and analysis of gene expression profiles from periodontal tissue biopsies are crucial for obtaining clear evidence in the specific diagnosis of periodontal disease. This diagnosis could be complex for early practitioners during routine examinations, and the use of ML techniques can be highly productive in such cases. Most of these studies used SVM as the classifier in the analysis [63-65,67]. Two of the studies [64,67] used other algorithms such as naïve Bayes, ANNs, and decision tree algorithms. A decision tree provides a hierarchical organization from a root node, which is at the highest hierarchical level. It is commonly argued that the decision tree has high explanatory capacity and transparency, and also provides comprehensibility in the analysis [60]. Several authors have made suggestions to further improve the effectiveness and simplicity of the models [68,69]. Further, the use of ensemble methods makes it is possible to construct more robust tree models, such as bootstrap aggregated (or bagged) decision trees, random forest trees, and boosted trees. In the case of naïve Bayes, it is considered simple and computationally efficient and uses all available information to explain the decision, which is an advantage in the clinical environment [60].

**Table 2:** Main features of surveyed studies using machine learning algorithms in periodontics

Dentistry field	Application	Data set	Machine learning algorithms	Performance
Periodontics	Diagnosis and classification of periodontitis based on the molecular profile Kebschull *et al*. [63] (2013)	Gene expression profiles of the entire genome from 310 biopsies of “healthy” or “sick” gingival tissue	SVM^a^	AUC^b^: 0.63-0.99
	
	Diagnosis of aggressive periodontitis and chronic periodontitis trained by immunologic parameters Papantonopoulos *et al*. [42] (2014)	4 distinct samples of patient with periodontitis advanced obtained from previous studies	ANNs^c^	Accuracy: 90-98%
	
	Diagnosis of periodontal diseases (preliminary study) Ozden *et al*. [64] (2015)	150 patients	SVM, DT^d^, and ANNs	Accuracy: SVM, 98%; DT, 98%; ANNs, 46%
	
	Classification of generalized chronic periodontitis, generalized aggressive periodontitis and periodontal health from bacterial profiles Feres *et al*. [65] (2018)	3915 subgingival biofilm samples from 435 patients SVM	SVM	Se^e^: 86%, Sp^f^:79%, AUC: 0.83
	
	Detection and classification of the periodontal bone loss of each individual tooth Chang *et al*. [66] (2020)	340 panoramic radiographs	CNNs^g^	ICC^h^: 0.91; *P*<0.01
	
	Periodontitis risk assessment Shimpi *et al*. [67] (2020)	11048 patients (4766 positive cases and 6282 controls)	Naïve Bayes, Logistic Regression, SVM, ANNs, and DT	Accuracy: Naïve Bayes, 0.801 (0.791-0.811); Logistic Regression, 0.768 (0.778-0.795); SVM, 0.790 (0.780-0.799); ANNs, 0.841 (0.833-0.849); DT, 0.901 (0.892-0.908)

a SVM: Support vector machine;

b AUC: Area under receiver operating;

c ANNs: Artificial neural networks;

d DT: Decision tree; Se^e^: Sensibility; Sp

f :Specificity;

g CNN: Convolutional neural networks;

h ICC: Intraclass correlation coefficient.

Preliminary research applying ANNs and using abundant samples collected from previous studies shows evidence for the good performance of this classifier [42]. Models using ANNs have already demonstrated their effectiveness in other fields of Medicine [59,70].

### 3.3. Applications in oral medicine and maxillofacial surgery (Table 3)

In the last decade, the applications of ML-based diagnosis have been expanded to the segmentation and identification of maxillofacial cysts and other radiolucent lesions, as well as the diagnosis of other common oral diseases of growing interest in the oral medicine and maxillofacial surgery domain [23,24,43,44,71]. The gold standard for the final diagnosis of these lesions is a specific histopathological examination, which tends to be more invasive and entails greater cost and resources. Therefore, the advancement of new methodologies that lead to more simplified and precise diagnostic procedures requires special attention. Several studies have successfully applied the CNNs algorithm for this purpose [23,44,71]. One of the studies reported that the best performance was achieved by using SVM (96% accuracy) to classify dental periapical cysts and keratocysts in a set of 3D cone beam computed tomography (CBCT) images [43]. The study also explored other classifiers such as ANNs, k-nearest neighbors (k-NN), naïve Bayes, decision trees, and random forest. The application of the k-NN classifier is based on the principle that nearby data points have similar properties. Reports show that k-NN is very sensitive to irrelevant characteristics, which can make learning inefficient and affect the interpretation of the final model. However, the transparency of this classifier makes it useful, as it reflects the intuition of human users. This characteristic is common to naïve Bayes classifiers as well [72]. In general, the selection of these algorithms for specific objectives is a difficult task. Further, the evaluation of their performances may be different according to the characteristics and pre-processing of the datasets [60,72].

**Table 3:** Main features of surveyed studies using machine learning algorithms in oral medicine and maxillofacial surgery

Dentistry field	Application	Data set	Machine learning algorithms	Performance
Oral medicine and maxillofacial surgery	Diagnosis and classification of dental periapical cyst lesions and keratocystic odontogenic tumor through decision support system Yilmaz *et al*. [43] (2017)	50 CBCT^a^ 3D images obtained from 50 patients	k-NN^b^, Naive Bayes, DT^c^, Random forest , ANNs^d^, and SVM^e^	Best accuracy (SVM) : 96.0%
	
	Diagnosis and classification of radiolucent lesions in the mandible on panoramic radiographs Ariji *et al*.[23] (2019)	210 panoramic radiographs images with mandibular radiolucent lesions	CNNs^f^	Se^g^: 0.88
	
	Diagnosis for cases of orthognathic surgery Choi *et al*. [79] (2019)	316 cases	ANNs	Success rate (surgery/non-surgery diagnosis): 96% ICC^h^: 0.97- 0.99
	
	Develop an intelligent automated system to support specialists for differential diagnosis in oral medicine Ehtesham *et al*. [24] (2019)	500 cases of six principal axes of oral diseases	CBR^i^	Success rate: 76.9 %
	
	Predict perioperative blood loss prior to orthognathic surgery Stehrer *et al*. [78] (2019)	950 patients	Random forest	MD^j^:7.4 ml, SD^k^: 172.3 ml, p < 0.001
	
	Automatic classification of dental artifact status for efficient image veracity checks Welch *et al*. [51] (2019)	1538 head and neck planning CBCT images	CNNs	AUC^l^: 0.91±0.01
	
	Detection and segmentation of the mental foramen Kats *et al*. [74] (2020)	112 digital panoramic radiographs	CNNs	Precision: 71.13%, recall:68.24%
	
	Diagnosis of odontogenic cysts and tumors of both jaws on panoramic radiographs Kwon *et al*. [71] (2020)	1282 panoramic radiographs (350 dentigerous cysts, 302 periapical cysts, 300 odontogenic keratocysts, 230 ameloblastomas, and 100 normal jaws with no disease	CNNs	Se: 88.9%, Sp^m^: 97.2%, accuracy: 95.6%, and AUC: 0.94, (augmented data set)
	
	Diagnosis of odontogenic cystic lesions Lee *et al*. [44] (2020)	2126 images, including 1140 (53.6%) panoramic and 986 (46.4%) CBCT images	CNNs	AUC: 0.914, Se: 96.1%, Sp:77.1%
	
	Develop a method for automated dental segmentation of panoramic dental images for diagnostic purposes Lee *et al*. [75] (2020)	846 images with tooth annotations from panoramic radiographs	CNNs	F1-score: 0.875, precision: 0.858, recall: 0.893, IoU^n^:0.877
	
	Classification of head and neck computed tomography images to detect the presence of dental artifacts that affect the visualization of structures Welch *et al*. [76] (2020)	1538 computed tomography images	CNNs	AUC: 0.92±0.03
	
	Classification of maxillary sinus lesions compared to healthy maxillary sinuses Kuwana *et al*. [77] (2021)	Imaging data for healthy maxillary sinuses (587 sinuses), inflamed maxillary sinuses (416 sinuses), cysts of maxillary sinus regions (171 sinuses)		Accuracy, Se and Sp: , 90-91%, 88-85%, and 91-96% (diagnosis maxillary sinusitis); 97-100%, 80-100%, and 100% (cysts of the maxillary sinus regions)

a CBCT: Cone beam computed tomography;

b k-NN: k-nearest neighbors;

c DT: Decision tree;

d ANNs: Artificial neural networks;

e SVM: Support vector machine;

f CNN: Convolutional neural networks;

g Se: Sensibility;

h ICC: Intraclass correlation;

i CBR: Case-based reasonig;

j MD: Mean absolute difference;

k SD: Standard deviation;

l AUC: Area under receiver operating curve;

m Sp: Specificity;

n IoU: Intersection-over-union.

Other studies have reported the application of selection case-based reasoning (CBR) in the analysis [24]. CBR provides feedback through the collection of the previous cases and learns from these. Thus, new rules can be defined even with the addition of new cases. Many conditions of the oral cavity show similar clinical signs and symptoms, which can often hinder the diagnosis [24,73]. This affects the reliability of the diagnosis and the subsequent treatment scheme to be followed by the patient. The CBR technology has been valuable in developing a meticulous and systematic approach for the unique identification of these diseases, seeking a more exact characterization of analogies and features that distinguish them. The results, though incipient, highlight the capability of the algorithms in improving the quality and efficiency of patient care through accurate diagnosis.

These techniques have also been applied in the classification of maxillary sinus lesions, detection, and segmentation of structures, as well as classification of the state of dental artifacts for efficient image veracity checks [51,74-77].

The success of ML algorithms in predicting perioperative blood loss before orthognathic surgery has also been recently investigated [78]. The use of a random forest classifier could anticipate possible complications during the surgical procedures and calculate the likely perioperative blood loss before performing the surgery. This prediction could be useful in decision-making by professionals and patients in elective procedures and facilitate better management of surgical procedures. In a recent study, ANNs were used to determine the diagnosis of orthognathic surgery cases, showing a model success rate of 96% for diagnostic decisions [79].

#### 3.2.1. Applications in forensic dentistry (Table 4)

Forensic dentistry and anthropological examinations have also been impacted by the advances of these modern tools. One of the studies that aimed to classify tooth types based on dental CBCT images applied CNNs algorithms with an accuracy of 88.8% [80]. Several studies have focused on the application of automated methods in the estimation of age using teeth, an important aspect of forensic dentistry [48,81-83]. Manual methods that estimate age based on the stage of tooth development are painstaking and complex. An interesting study focused on the evaluation of skeletal patterns using automated algorithms [84]. Automated systems that contribute to improving the quality and speed of ML-based age estimation are very useful and warrant more attention devoted to their further development and validation [82].

**Table 4:** Main features of surveyed studies using machine learning algorithms in forensic dentistry

Dentistry field	Application	Data set	Machine learning algorithms	Performance
Forensic dentistry	Classification of tooth types on dental CBCT^a^ images for forensic identification purposes Miki *et al*. [80] (2017)	52 CBCT image	CNNs^b^	Accuracy (augmented training data): 88.8%
	
	Use of automated learning techniques for predicting mandibular morphology in skeletal class I, II and III Niño-Sandoval *et al*. [84] (2017)	229 lateral cephalograms (95 women and 134 men); age range 18-25 years	ANNs^c^, SVR^d^	ICC^e,:^ ANNs, 0.84 - 0.99; SVR, two coefficients above 0.7
	
	Age estimation in children and adolescents for forensic purposes Štepanovský *et al*. [81] (2017)	976 orthopantomographs (662 males, 314 females) of children and adolescents; age range 2.7-20.5 years	SVM^f^, ANNs, k-NN^g^, K-Star, Regression tree, M5P Tree, Random forest	MAE^h^: M5P tree, SVM: under 0.7 years for both males and females
	
	Develop an automated method for dental age estimation based on lower third molars stage on panoramic radiographs (pilot study) De Tobel *et al*. [82] (2019)	400 panoramic radiographs	CNNs	Mean accuracy: 0.51, MD^i^: 0.6 stages, kappa: 0.82
	
	Automatic human identification from panoramic dental radiographs Fan *et al*. [83] (2020)	15369 panoramic dental radiographs from 6300 individuals	CNNs	Accuracy: 85.16% and 97.74%
	
	Automated estimation of tooth age from lower left third molar development stages assessed on panoramic radiographs Merdietio *et al*. [48] (2020)	400 panoramic radiographs	CNNs	Accuracy: 0.61, MD: 0.53 stages, kappa: 0.84

a CBCT: Cone beam computed tomography;

b CNNs: Convolutional neural networks;

c ANNs: Artificial neural networks;

d SVR: Support vector regression;

e ICC: Intraclass correlation coefficient;

f SVM: Support vector machine;

g k-NN: k-nearest neighbors;

h MAE: Mean absolute error;

i MD: Mean absolute difference.

#### 3.2.2. Applications in endodontics (Table 5)

Recent studies employing these techniques in endodontics have indicated several performance advantages. However, some of these studies constitute pre-clinical studies and the generalization of their results has been limited. Among the applications in this area, locating the minor apical foramen using feature extraction procedures from radiographs is distinguished [85,86]. These *ex vivo* studies applied ANNs and revealed promising results that should be further explored. The exact estimation of the working length is an important initial step to a successful root canal treatment. The introduction of improvements in locating the area of greatest constriction (minor apical foramen) in the root canal constitutes a key element of renewed interest for clinical endodontists.

**Table 5:** Main features of surveyed studies using machine learning algorithms in endodontics and cariology

Dentistry field	Application	Data set	Machine learning algorithms	Performance
Endodontics	Localization of the lesser apical foramen using X-ray feature extraction procedures and then processing data using an artificial neural network as a decision-making system Saghiri *et al*. [85] (2012)	50 single-rooted mandibular incisors and second premolars with curvatures <30° in the apical region, extracted for orthodontic or periodontal reasons	ANNs^a^	Success rate: 93%
	
	Location of the minor apical foramen using feature extraction procedures from radiographs (a cadaver study) Saghiri *et al*. [86] (2012)	50 single-rooted teeth from 19 male cadavers; age range 49-73 years	ANNs	Success rate: 96%, CI^b^: 90.57–101.43; P<0.001
	
	Diagnosis of vertical root fracture (an ex vivo study) Kositbowornchai *et al*. [87] (2013)	200 digital radiographic images of extracted human premolar teeth with single root (50 sound and 150 vertical root fractures)	ANNs	Se^c^: 98%, Sp^d^: 90.5%, accuracy: 95.7%
	
	Diagnosis of the number of the distal roots of mandibular first molars Hiraiwa *et al*. [88] (2019)	Panoramic radiographs of 760 mandibular first molars from 400 patients	CNNs^e^	Accuracy:86.9%
	
	Detection of vertical root fracture on panoramic radiograph using neural network system Fukuda *et al*. [89] (2019)	300 panoramic images containing a total of 330 teeth with clearly visible fracture lines	CNNs	Recall: 0.75, precision: 0.93, F-measure: 0.83
	
	Diagnosis of periapical pathosis on CBCT^f^ images Orhan *et al*. [49] (2020)	Images of 153 periapical lesions obtained from 109 patients	CNNs	Recall: 0.89, precision:0.95, F-measure: 0.93

Cariology	Approximal caries diagnosis using a computer-assisted diagnostic system Araki *et al*. [26] (2010)	100 approximate surfaces of 50 human teeth extracted (first and second maxillary premolars)	ANNs	AUC^g::^ Inner half enamel caries, 0.691±0.048; dentine caries, 0.745±0.088; all caries, 0.662±0.061
	
	Diagnosis of dental caries on periapical radiographs Lee *et al*. [27] (2018)	3000 periapical radiographic images	CNNs	Premolar and molar model-Se: 81.0 % (74.5-86.1), Sp: 83.0 % (76.5-88.1), accuracy: 82.0% (75.5-87.1), PPV^h^: 82.7% (76.1-87.9), NPV^i^: 81.4 (75.0-86.4), AUC: 0.845 (95% CI 0.790-0.901)
	
	Develop models for identification of root caries risk Hung *et al*. [28] (2019)	5135 individuals; mean age (±SD^j^): 46,6 ± 18,1 years	SVM^k^, XGBoost^l^, Random forest, k-NN^m^, LR^n^	Se, Sp, accuracy, precision, AUC: SVM: 0.996, 0.943, 0.97, 0.951, 0.997; XGBoost: 1.000, 0.889, 0.947, 0.908, 0.987; Random forest:1.000, 0.875, 0.941, 0.947, 0.999; k?NN: 0.971, 0.679, 0.832, 0.769, 0.881; LR: 0.771, 0.711, 0.742, 0.742, 0.818
	
	Detection of caries lesions on bitewing radiographs images Cantu *et al*. [29] (2020)	3686 bitewing radiographs	CNNs	Se:0.75, Sp: 0.83, accuracy: 0.80, PPV: 0.70, NPV: 0.86, F-measure: 0.73
	
	Diagnosis of dental caries on digital periapical radiographs Geetha *et al*. [30] (2020)	105 images derived from intra-oral digital radiography	BPNN^o^	Accuracy: 0.971, AUC: 0.987
	
	Caries risk prediction in geriatric people Liu *et al*. [31] (2020)	1144 geriatrics; range age 65-74 years	GRNN^p^	Se: 91.41% (training set), 85.16% (test set); Sp: 72.38% (training set), 70.27% (test set); AUC: 0.777
	
	Detect caries lesions in near-infrared-light transillumination images Schwendicke *et al*. [32] (2020)	226 extracted posterior permanent human teeth (113 premolars, 113 molars)	CNNs	AUC: 0.74 (0.66-0.82), Se: 0.59 (0.47-0.70), Sp: 0.76 (0.68-0.84), PPV: 0.63 (0.51-0.74), NPV: 0.73 (0.65-0.80)
	Evaluation of cost effectiveness in detection of proximal caries on bitewing radiographs using AI Schwendicke *et al*. [90] (2020)	3686 bitewing radiographs	CNNs	Accuracy: 0.80; *P*<0.05, ICER^q^: –13.9 euro/year

a ANN: Artificial neural networks;

b CI: Confidence interval;

c Se: Sensibility;

d Sp: Specificity;

e CNN: Convolutional neural networks;

f CBCT: Cone beam computed tomography;

g AUC: Area under receiver operating;

h PPV: Positive predictive value;

i NPV: Negative predictive value;

j SD: Standard deviation;

k SVM: Support vector machine;

l XGBoost: Extreme gradient boosting;

m k-NN: k-nearest neighbors;

n LR: Logistic regression;

o BPNN: Back propagation neural networks;

p GRNN: General regression neural network;

q ICER: Incremental cost-effectiveness ratio.

Another study aimed at evaluating the performance of ANNs in the diagnosis of vertical root fracture using a moderate sample of extracted teeth [87]. Although the results were successful, a larger number of teeth and other dental groups should be included in the analysis to arrive at more reliable conclusions. The previous studies that collected *in vivo* data were also reviewed [49,88,89]. These studies used CNNs algorithms to aid in the diagnosis of the number of roots of the mandibular first molars [88], to detect vertical root fractures on panoramic radiographs [89], and to improve the diagnosis of periapical pathosis on CBCT images [49]. One of the studies [88] found that deep learning systems using CNNs to process inexpensive radiographic images showed high accuracy (86.9%) in the differential diagnosis of a single or extra root in the distal roots of the mandibular first molars.

#### 3.2.3. Applications in cardiology (Table 5)

Findings from previous studies already reflect possible applications, both for the development of diagnostic systems for caries lesions through images and for the estimation of the prognosis of the disease [26-32,90]. Furthermore, dental caries continues to be a major oral health problem for many groups of the population [91-93]. In this context, new alternatives and tools that guarantee improvements in current methods of diagnosis and prognosis will be well-received among practicing dentists and patients.

One particular study [28] aimed to develop models for the prediction of root caries in adults and reported an excellent performance from a large database. Among the algorithms used, the SVM-based method demonstrated the best performance for the identification of root caries, with an accuracy of 97.1%, precision of 95.1%, sensitivity of 99.6%, and specificity of 94.3%. However, the study reported some problems concerning the use of cross-sectional data, which could affect the predictive value of the model. Generalization and validation strategies using longitudinal data should be further explored. Another study, which stands out for the quality of its design, focused on the prediction of caries in geriatric patients using general regression neural networks (GRNN), and showed promising results, with a sensitivity of 91.41% on the training set and 85.16% on the test set [31]. GRNN represents an improved neural network technique based on nonparametric regression. These algorithms can be very useful for making predictions and comparisons of system performance in practice due to their ability to converge upon the underlying function of the data with only a few training samples. The additional knowledge required to obtain the adjustment successfully is relatively small and can be achieved without additional input from the user, which is highly advantageous [31,37].

A novel study evaluated, for the 1^st^ time, the cost-effectiveness of these technologies, which is a vital consideration for their application in the clinical environment [90]. The study reported encouraging results for the use of automated methods in the diagnosis of caries.

### 3.3. Applications in prosthetics, conservative dentistry, and implantology (Table 6)

Other subfields of dentistry such as prosthetics, conservative dentistry, and implantology have also benefited from the implementation of ML techniques. The use of ANN-based systems that support the prognosis of facial deformation after a complete prosthesis has been promoted in these fields. The experimental results showed that this method can predict the deformation of facial soft tissues quickly and accurately, which is valuable in making decisions to establish concrete treatment actions [94]. Other studies have focused on the classification of specific features of teeth using ANNs [95], the potential of ANNs in improving tooth color through computer-assisted systems [96], and the automated detection and classification of various dental restorations in panoramic radiographs using SVM [97]. The study using SVM reported high accuracy (93.6%).

**Table 6:** Main features of surveyed studies using machine learning algorithms in prosthetics, conservative dentistry and implantology

Dentistry field	Application	Data set	Machine learning algorithms	Performance
Prosthetics, conservative dentistry and implantology	Prediction of facial deformation after complete denture prosthesis Cheng *et al*. [94] (2015)	Preoperative and postoperative 3D face scan of 48 patients ANNs^a^	ANNs	Average error rate: 22.49%
	
	Classification of specific characteristics of teeth based on 3D scan data Raith *et al*. [95] (2017)	129 data sets, consisting of 69 upper jaw virtual models and 60 lower jaw virtual models	ANNs	Success rate: 93.3% and 93.5%
	
	Prediction of patient mean peri-implant bone level Papantonopoulos *et al*. [101] (2017)	72 implant-treated patients with 237 implants (mean 7.4±3.5 years of function)	SVM^b^	Se^c^: 55% , Sp^d^: 91%, particle swarm optimization-SVM: Se: 62%, Sp: 85%
	
	Improve the computer color matching system (CCM) to measure a tooth color quantitatively and offer a porcelain recipe with instructions Wei *et al*. [96] (2018)	43 metal-ceramic specimen	ANNs	MD^e^: 1.89 ± 0.75; P<0.01
	
	Predicting the debonding probability of a computer-aided design/computer-aided manufacturing composite resin crowns with 3D stereolithography models of a die scanned from patients Yamaguchi *et al*. [98] (2019)	8640 images	CNNs^f^	Accuracy: 98.5%, precision: 97.0%, recall: 100%, F-measure: 0.985, AUC^g^: 0.998
	
	Automatic detection and classification of various dental restorations on panoramic radiographs Abdalla-Aslan *et al*. [97] (2020)	738 dental restorations in 83 anonymized panoramic images	SVM	Overall accuracy: 93.6%
	
	Construct a clinical decision support model to predict tooth extraction therapy in clinical situations by using electronic dental records Cui *et al*. [99] (2020)	4135 unidentified electronic dental records from 3559 patients	Regression tree algorithm, AdaBoost^h^, GBDT^i^, Light GBM^j^, and XGBoost^k^	Accuracy, AUC^l^: regression tree algorithm: 0.953, 0.919; AdaBoost: 95;5, 0.970; GBDT: 0.957, 0.970; LightGBM: 0.957, 0.970; XGBoost: 0.962,0.970
	
	Identification of the brand and model of a dental implant from radiographs Hadj Saïd *et al*. [100] (2020)	1206 dental implant radiographic images	CNNs	Accuracy: 93.8% (95% CI^m^: 87.2-99.4%), Se: 93.5% (95% CI: 84.2-99.3%), Sp: 94.2% (95% CI: 83.5-99.4%), PPV^n^: 92% (95% CI: 83.9-97.2%), NPV^o^: 91.5% (95% CI: 80.2-97.1%), AUC: 0.918 (95% CI: 0.826-0.973)
	
	Identification and classification of dental implant systems, using panoramic and periapical radiographs (a pilot study) Lee *et al*. [45] (2020)	5390 panoramic and 5380 periapical radiographic images from 3 types of dental implant systems	CNNs	AUC: 0.971, 95% CI: 0.963-0.978

a ANN: Artificial neural networks;

b SVM: Support vector machine;

c Se: Sensibility;

d Sp: Specificity;

e MD: Mean absolute difference;

f CNN: Convolutional neural networks;

g AUC: Area under receiver operating;

h AdaBoost: Adaptive boosting;

i GBDT: Gradient boosting decision tree;

j Light GBM: Light gradient boosting machine;

k XGBoost: Extreme gradient boosting;

l AUC: Area under receiver operating curve;

m CI: Confidence interval;

n PPV: Positive predictive value;

o NPV: Negative predictive value.

Other applications focused on using CNNs to predict the probability of shedding composite resin crowns fabricated with computer-aided design [98]. Another study used the Extreme Gradient Boost (XGBoost) algorithm to develop a clinical decision support model for the prediction of tooth extraction therapy related to the subsequent use of dentures [99]. The algorithm showed an accuracy of 96.2% and proved to be a powerful classifier and regressor, generally reaching optimal performance in structured data.

In the field of implantology, two studies demonstrated a good performance of CNNs in detecting implant systems [45,100]. The placement of dental implants as a branch of rehabilitation treatment has recently become common among patients who require more aesthetically and functionally sound dental prosthetic rehabilitation. At present, a considerable number of implant systems are placed with different fixation structures and characteristics, such that their identification based on routine radiographic images can be complicated for clinical dentists. Proper identification of these systems would reduce more invasive treatments in which the patient requires some type of repair or repositioning of the implant system. Another application in this area is focused on the prediction of patients’ mean peri-implant bone levels, which is very useful for estimating the survival of the implant and exploring plausible treatment alternatives that lead to the best outcomes [101]. This study used SVM for modeling and reported moderate performance.

## 4. Key Factors Influencing the Clinical Implementation of ML

The findings presented above point to a promising future of ML algorithms applied in dental practice. In this section, we will outline some key factors that should be considered to effectively guide the practical application of these methodologies.

### 4.1. Defining the clinical uncertainty

A clear definition of specific clinical uncertainty must be identified. The desired outcomes should be properly defined to guide the research. In addition, the objective should be adequately represented in terms of inputs and outputs, must correspond to plausible criteria, and consider the real clinical scenario in addition to results of other analyses. Reduction of heterogeneity between reports should be ensured with the use of clear and unambiguous guidelines. A previous study makes valuable suggestions in this regard [102].

### 4.2. Data management

Reproducing the findings of developmental studies in clinical practice poses certain challenges. Studies using ML attempt to generalize the links of input and output variables in the data set through the learning process. However, data sets are often confounded by noise. Therefore, the intrinsic characteristics of the data (e.g. categorical data, numeric data, and time-series), origin, volume, outliers, missing data, etc., could affect the reliability of the models and lead to false interpretations while evaluating performance [39]. For example, variations in classification criteria of periodontal disease (target condition) were detected when retrospective data were analyzed. Further, one must be aware of what is called “data leakage” [8,39,40]. Data leakage occurs when a certain attribute accidentally encodes the result (e.g. when the need for a partial denture already indicates the diagnosis of edentulism). Initially, covariates with the same meaning must be eliminated. Criteria on biological plausibility should guide the selection of covariates. Strategies to reduce data dimensionality are fundamental in ensuring simplicity and effectiveness of the models. Thus, data pre-processing, cleaning, and normalization are essential steps in all analyses [39,103]. Automated techniques such as constructive induction, attribute interaction discovery, and non-linear modeling approaches through embedded, filters or wrappers methods have been used for data mining [104,105]. One of the reviewed studies that aimed to estimate predictors associated with peri-implantitis implemented some of these techniques in an attempt to reduce the dimensionality of the data [101].

Moreover, biases can be introduced when the data do not capture the epidemiological reality of events [40]. For example, a dental caries prediction model built on specific attributes of a population of adolescent dental students from high-income countries may not be generalizable to make diagnostic inferences of periodontal disease for all the population, without including women in the initial training. These predictions would introduce certain disparities and false interpretations in the models. In this context, it is essential to collect representative samples based on idealized hypotheses. The use of traditional power calculation methods, considering the size of important clinical differences, similarity, non-inferiority, or superiority of the models using ML in contrast with the gold standard, may allow for later extrapolation of the results [106]. A previous study focused on the prediction of dental caries better illustrates this analysis [31].

Emphasis should be placed on the availability of data from real examples for subsequent application of the model in clinical practice [39,106]. Public access repositories that record specific medical and dental data could be very useful for achieving this goal [40]. One of the previous studies made use of public-use data from the National Health and Nutrition Examination Survey and reported good performance of ML classifiers in predicting root caries in this dataset [28]. Repositories with specific dental information are scarce. In this sense, efforts to create interoperable data sources should be expanded to provide support for the implementation of these methodologies [39]. This will, in turn, help in the evaluation of their performance by achieving more transparency and reproducibility in reporting. We should note the variability related to differences in annotations and particular characteristics of the data sources (medical records, X-rays, photographs, laboratory tests, and models) between various repositories [39]. Therefore, we consider it essential to apply standards to AI-based technologies, creating a common nomenclature that facilitates the implementation of consistent methods of data storage and retrieval across public platforms. Moreover, dental records and medical reports should be preserved and submitted to expert committees to integrate into these repositories [107,108].

### 4.3. Training, validation, and test sets

Given the lack of certainty regarding the algorithm to obtain the best classifier, it is useful to have non-overlapping, random datasets for training, validation, and/or testing. Thus, comparisons between different algorithms can be made on the training set, the model can be tuned and optimized on the validation set, and the performance of the model can be evaluated on the test set [108]. This process reduces the possibility of over fitting the model conditioned by the memorization of the characteristics of the training data, which can cause the model to fail when used in an independent sample. Similarities in the success rates of the training set, validation set, and test set imply the best generalization of the model. A study that used neural networks in the diagnosis of teeth extractions followed by the above approach for evaluating the capacity of expert systems with ML showed adequate performance for the different sets. Thus, the system could be tested more extensively to support decision-making during dental practice [19]. Other approaches to guide validation, such as resampling methods (e.g. bootstrapping and cross-validation), have been heavily recommended in clinical prediction model guidelines, when is not possible to obtain a separate set [4,109,110]. However, the results should be interpreted with caution as only the best results were reported while comparing several algorithms using this procedure.

As the number of examples influences the learning process in ML, some strategies are expected to contribute to the improvement of training. Augmentation can be used when the image dataset is collected. This technique allows us to artificially expand the size of a dataset by creating modified versions of images, improving the ability of fit models to generalize what they have learned to new images. The use of transfer learning can also provide training opportunities and help improve model performance. Several previous dental studies have implemented these approaches [27,29,56,88]. Over-sampling and repeat-sampling techniques can also be applied to reduce biases and improve model performance [106,110].

### 4.4. External validation

Even when internal validation is applied reserving a separate set of the same sample for testing, training data would be too personalized and the performance of the classifiers could be overestimated. Therefore, to extrapolate the results and ensure certainty in the clinical performance of the models, verification through an independent set (external validation) is crucial. External validation should be done in cohort studies, ideally with data acquired independently by means of geographic (e.g. dataset collected from a department of pediatric dentistry in another district) or temporal (e.g. dataset collected from other pediatric patients in the same department after 6 months) splits. Open access data sets, if available, could also be obtained [39,106].

### 4.5. Unbalanced classes

One of the recurring problems encountered during medical diagnosis is facing unbalanced classes where there is a disproportionate number of observations in each class (e.g. caries present or caries absent; oral cancer or not oral cancer). In this scenario, the classifiers exhibit poor precision in the minority class, as standard algorithms are designed to maximize accuracy and reduce error rates. They ignore the difference between types of misclassification errors, which can prove costly in medical or dental practice [111]. For example, classification in the diagnosis of oral cancer may favor false negatives (individuals with cancer but classified as negative). Consequently, these patients would not receive medical care. In this scenario, the accuracy metric may show a high value but bring a misleading understanding of the actual performance of the model. Solutions for this problem have been suggested both at the data level and at the algorithmic level. Solutions at the data level include different ways of resampling such as random oversampling with replacement, random under sampling, directed oversampling (where the choice of samples to replace is reported, rather than random), directed under sampling (where the choice of examples to be eliminated is reported), oversampling with informed generation of new samples, or combinations of the above techniques. Solutions at the algorithmic level include adjusting costs of the various classes to counteract the class imbalance; for example, decision trees can be used to adjust the probabilistic estimate on the tree leaf [111,112].

### 4.6. Reference standard (ground truth)

In the field of data mining, the definition of the standard reference is important for labeling the data and evaluating the performance of the classifier. Arriving at an optimal definition can be challenging. The clinical reference standard is understood as the best available method to establish the presence or absence of the target condition. However, the gold standard would be an error-free reference standard [113]. Consequently, we can deduce that these reference standards are often not perfect. A concrete example is the routine visual and tactile examination used by the clinician for the diagnosis of dental caries, which can lead to errors. In this context, sensitivity or specificity values may be overestimated or underestimated. Evaluating the correlation between the performance of the classifier and the standard reference is necessary, and the use of external data may generate new adjustments. Assuming a composite reference standard that combines the results of multiple imperfect tests may be another alternative. Routinely used clinical data may add additional value to the analyses [114]. For example, when evaluating the presence of periodontitis, clinical observations, such as profiling of the pocket on probing, signs and symptoms of the periodontium, and bleeding, should be considered in addition to considering the bone level using radiographic criteria. This will provide a more robust standard and reduce possible misinterpretation. Panel or consensus diagnosis including a sufficiently large sample of experts could generate greater credibility compared to the isolated judgment of an expert. The inclusion of less experienced clinicians should be limited, as it can lead to errors in the interpretation of the algorithms’ performance. In addition, the calibration process and methods to measure inter- and intra-variability should be considered [115].

### 4.7. Classifier selection

The selection of the appropriate classifier still constitutes a gap in the literature related to the topic. Most of the classifiers proposed in the studies reviewed by us showed good performance. In order to extend these studies to clinical dental practice, interpretable models are preferred. These models allow for a better explanation of the selected parameters and will be more useful in decision-making [110]. Therefore, if these classifiers show similar precision levels while comparing several models, the explainable ones should be prioritized. Accuracy results can be misleading, especially when using unbalanced classes. Metrics such as the confusion matrix and area under the receiver operating characteristic curve (AUC) can provide better insights into model performance in clinical practice [103,106,116]. Other metrics for evaluating models used for the detection or segmentation of objects in images have also been reported [102].

### 4.8. Deployment and clinical application

Some aspects should be carefully evaluated before extending the application of these technologies to clinical practice. As with their usefulness in decision-making, cost-effectiveness analysis, benefits in the quality of services, and acceptance of their introduction by patients and clinicians ought to be considered. However, studies on the practice usefulness of models using data mining in dentistry are rare [117]. A recent report that provides details in relation to the evaluation of cost-effectiveness in the detection of proximal caries supported the possibility of introducing these tools in clinical practice [90]. In general, it is necessary to check out-of-sample datasets to verify the usefulness of these technologies before their dissemination in dental practice. Well-designed observational studies should focus on evaluating the impacts of these tools over time, by comparing scenarios wherein these tools have been used with those wherein they have not been used. The literature reviewed by us lacks randomized clinical trials, which should also be encouraged to improve the body of evidence on the potential for data mining in dental practice [106,117]. Moreover, studies conducted across institutions may better reflect the capabilities of these technologies and give greater external validity to the results [39].

Another key point is that these analyses are often reserved for specialists in an area with no appropriate interface provided for the implementation of these resources by routine health professionals. The interdisciplinary scientific community should perpetuate innovative solutions to contribute to improvements in this area. In dentistry, our findings revealed the development of several automated systems that have shown good performances, especially when applied to image-based diagnosis. Exploratory studies with a focus on the aforementioned aspects will be able to guarantee the practical expansion of these technologies in the clinical setting.

Further, the community of health professionals should also offer formal acceptance of use in service after rigorous analysis of these technologies to guarantee the effectiveness of their application. These technologies will also be a source of data storage for future research and evaluation of the best evidence related to certain diagnosis or treatment schemes [8]. However, the utility of these systems will not replace the function of the clinician, who generally evaluates other aspects in relation to the clinical, psychological, and behavioral conditions of the patient. Rather, it will serve to facilitate certain decisions in the real environment. The financial aspects of the implementation of ML are usually another concern. As these techniques undergo improvements with their continued use in healthcare, greater financial contributions will be required for research and further development [107,116,118].

## 5. Final Considerations

The use of ML has recently been expanded to different clinical dental specialties. Algorithms such as CNNs and SVM have shown promise in boosting the future use of these resources in different aspects of dental clinical practice. They offer a vast arsenal of tools to support diagnosis and prognosis and to improve clinical decisions. The ethical aspects of accessing and handling large amounts of sensitive information deserve special attention. Meticulous data pre-processing is recommended to extract useful models. The use of longitudinal study designs and verification through clinical trials should be guided by the advances of these novel techniques. External validation must also be extended to guarantee the use of efficient and generalizable models. In addition, the homogenization of the methodologies for the presentation of these studies in clinical practice should be systematically improved for a better understanding of such proposals. Future studies in the field of dentistry should be aimed at obtaining solid evidence on the performance of these models and promoting their introduction in general practice.

## References

[1] Turing IB (1950). Computing Machinery and Intelligence. Am Turing Mind.

[2] Tegmark M (2017). Life 3.0:Being Human in the Age of Artificial Intelligence.

[3] Ahuja AS (2019). The Impact of Artificial Intelligence in Medicine on the Future Role of the Physician. PeerJ.

[4] Hung K, Montalvao C, Tanaka R, Kawai T, Bornstein MM (2020). The Use and Performance of Artificial Intelligence Applications in Dental and Maxillofacial Radiology:A Systematic Review. Dentomaxillofac Radiol.

[5] Collen MF, Shortliffe EH (2015). The creation of a new discipline. The History of Medical Informatics in the United States.

[6] Park WJ, Park JB (2018). History and Application of Artificial Neural Networks in Dentistry. Eur J Dent.

[7] Ramesh AN, Kambhampati C, Monson JR, Drew PJ (2004). Artificial Intelligence in Medicine. Ann R Coll Surg Engl.

[8] Rajkomar A, Dean J, Kohane I (2019). Machine Learning in Medicine. N Engl J Med.

[9] Wiens J, Campbell WN, Franklin ES, Guttag JV, Horvitz E (2014). Learning data-driven patient risk str. Jpegication models for clostridium difficile. Vol. 1. Open Forum Infectious Diseases.

[10] Henry KE, Hager DN, Pronovost PJ, Saria S (2015). A Targeted Real-time Early Warning Score (TREWScore) for Septic Shock. Sci Transl Med.

[11] Colubri A, Silver T, Fradet T, Retzepi K, Fry B, Sabeti P (2016). Transforming Clinical Data into Actionable Prognosis Models:Machine-learning Framework and Field-deployable App to Predict Outcome of Ebola Patients. PLoS Negl Trop Dis.

[12] Gulshan V, Peng L, Coram M, Stumpe MC, Wu D, Narayanaswamy A (2016). Development and Validation of a Deep Learning Algorithm for Detection of Diabetic Retinopathy in Retinal Fundus Photographs. JAMA.

[13] Motwani M, Dey D, Berman DS, Germano G, Achenbach S, Al-Mallah MH (2017). Machine Learning for Prediction of All-cause Mortality in Patients with Suspected Coronary Artery Disease:A 5-year Multicentre Prospective Registry Analysis. Eur Heart J.

[14] Kehl KL, Elmarakeby H, Nishino M, Van Allen EM, Lepisto EM, Hassett MJ (2019). Assessment of Deep Natural Language Processing in Ascertaining Oncologic Outcomes from Radiology Reports. JAMA Oncol.

[15] Morgan DJ, Bame B, Zimand P, Dooley P, Thom KA, Harris AD (2019). Assessment of Machine Learning vs Standard Prediction Rules for Predicting Hospital Readmissions. JAMA Netw.

[16] Leonardi RM, Giordano D, Maiorana F, Greco M (2010). Accuracy of Cephalometric Landmarks on Monitor-displayed Radiographs with and Without Image Emboss Enhancement. Eur J Orthod.

[17] Xie X, Wang L, Wang A (2010). Artificial Neural Network Modeling for Deciding if Extractions Are Necessary Prior to Orthodontic Treatment. Angle Orthod.

[18] Moghimi S, Talebi M, Parisay I (2012). Design and Implementation of a Hybrid Genetic Algorithm and Artificial Neural Network System for Predicting the Sizes of Unerupted Canines and Premolars. Eur J Orthod.

[19] Jung SK, Kim TW (2016). New Approach for the Diagnosis of Extractions with Neural Network Machine Learning. Am J Orthod Dentofac Orthop.

[20] Wang X, Cai B, Cao Y, Zhou C, Yang L, Liu R (2016). Objective Method for Evaluating Orthodontic Treatment from the Lay Perspective:An Eye-tracking Study. Am J Orthod Dentofac Orthop.

[21] Patcas R, Bernini DA, Volokitin A, Agustsson E, Rothe R, Timofte R (2019). Applying Artificial Intelligence to Assess the Impact of Orthognathic Treatment on Facial Attractiveness and Estimated Age. Int J Oral Maxillofac Surg.

[22] Kunz F, Stellzig-Eisenhauer A, Zeman F, Boldt J (2020). Artificial Intelligence in Orthodontics. J Orofac Orthop Fortschritte Kieferorthop.

[23] Ariji Y, Yanashita Y, Kutsuna S, Muramatsu C, Fukuda M, Kise Y (2019). Automatic Detection and Classification of Radiolucent Lesions in the Mandible on Panoramic Radiographs Using a Deep Learning Object Detection Technique. Oral Surg Oral Med Oral Pathol Oral Radiol.

[24] Ehtesham H, Safdari R, Mansourian A, Tahmasebian S, Mohammadzadeh N, Pourshahidi S (2019). Developing a New Intelligent System for the Diagnosis of Oral Medicine with Case-based Reasoning Approach. Oral Dis.

[25] Tamaki Y, Nomura Y, Katsumura S, Okada A, Yamada H, Tsuge S (2009). Construction of a Dental Caries Prediction Model by Data Mining. J Oral Sci.

[26] Araki K, Matsuda Y, Seki K, Okano T (2010). Effect of Computer Assistance on Observer Performance of Approximal Caries Diagnosis Using Intraoral Digital Radiography. Clin Oral Investig.

[27] Lee JH, Kim DH, Jeong SN, Choi SH (2018). Detection and Diagnosis of Dental Caries Using a Deep Learning-based Convolutional Neural Network Algorithm. J Dent.

[28] Hung M, Voss MW, Rosales MN, Li W, Su W, Xu J (2019). Application of Machine Learning for Diagnostic Prediction of Root Caries. Gerodontology.

[29] Cantu AG, Gehrung S, Krois J, Chaurasia A, Rossi JG, Gaudin R (2020). Detecting Caries Lesions of Different Radiographic Extension on Bitewings Using Deep Learning. J Dent.

[30] Geetha V, Aprameya KS, Hinduja DM (2020). Dental Caries Diagnosis in Digital Radiographs Using Back-propagation Neural Network. Health Inf Sci Syst.

[31] Liu L, Wu W, Zhang SY, Zhang KQ, Li J, Liu Y (2020). Dental Caries Prediction Based on a Survey of the Oral Health Epidemiology among the Geriatric Residents of Liaoning, China. Biomed Res Int.

[32] Schwendicke F, Elhennawy K, Paris S, Friebertshäuser P, Krois J (2020). Deep Learning for Caries Lesion Detection in Near-infrared Light Transillumination Images:A Pilot Study. J Dent.

[33] Tandon D, Rajawat J (2020). Present and Future of Artificial Intelligence in Dentistry. J Oral Biol Craniofac Res.

[34] Khanagar SB, Al-Ehaideb A, Maganur PC, Vishwanathaiah S, Patil S, Baeshen HA (2020). Developments, Application, and Performance of Artificial Intelligence in Dentistry-a Systematic Review. J Dent Sci.

[35] Schwendicke F, Golla T, Dreher M, Krois J (2019). Convolutional Neural Networks for Dental Image Diagnostics:A Scoping Review. J Dent.

[36] Jordan MI, Mitchell TM (2015). Machine Learning:Trends, Perspectives, and Prospects. Science.

[37] Michie D, Spiegelhalter DJ, Taylor CC (1994). Machine Learning. Neural Stat Class.

[38] Alpaydin E (2020). Introduction to Machine Learning.

[39] Wiens J, Shenoy ES (2018). Machine Learning for Healthcare:On the Verge of a Major Shift in Healthcare Epidemiology. Clin Infect Dis.

[40] Ngiam KY, Khor W (2019). Big Data and Machine Learning Algorithms for Health-care Delivery. Lancet Oncol.

[41] Leite AF, Vasconcelos KD, Willems H, Jacobs R (2020). Radiomics and Machine Learning in Oral Healthcare. Proteomics Clin Appl.

[42] Papantonopoulos G, Takahashi K, Bountis T, Loos BG (2014). Artificial Neural Networks for the Diagnosis of Aggressive Periodontitis Trained by Immunologic Parameters. PLoS One.

[43] Yilmaz E, Kayikcioglu T, Kayipmaz S (2017). Computer-aided Diagnosis of Periapical Cyst and Keratocystic Odontogenic Tumor on Cone Beam Computed Tomography. Comput Methods Prog Biomed.

[44] Lee JH, Kim DH, Jeong SN (2020). Diagnosis of Cystic Lesions Using Panoramic and Cone Beam Computed Tomographic Images Based on Deep Learning Neural Network. Oral Dis.

[45] Lee JH, Jeong SN (2020). Efficacy of Deep Convolutional Neural Network Algorithm for the Identification and Classification of Dental Implant Systems, Using Panoramic and Periapical Radiographs:A Pilot Study. Medicine.

[46] Zhu X, Goldberg AB (2009). Introduction to Semi-supervised Learning. Synthe Lectures Artif Intel Mach Learn.

[47] Deo RC (2015). Machine Learning in Medicine. Circulation.

[48] Merdietio Boedi R, Banar N, De Tobel J, Bertels J, Vandermeulen D, Thevissen PW (2020). Effect of Lower Third Molar Segmentations on Automated Tooth Development Staging Using a Convolutional Neural Network. J Forensic Sci.

[49] Orhan K, Bayrakdar IS, Ezhov M, Kravtsov A, Özy Özy TA (2020). Evaluation of Artificial Intelligence for Detecting Periapical Pathosis on Coneg Using a Convolutional Neural NeInt. Endod J.

[50] Vranckx M, Van Gerven A, Willems H, Vandemeulebroucke A, Ferreira Leite A, Politis C (2020). Artificial Intelligence (AI)-Driven Molar Angulation Measurements to Predict Third Molar Eruption on Panoramic Radiographs. Int J Environ Res Public Health.

[51] Welch ML, McIntosh C, Traverso A, Wee L, Purdie TG, Dekker A (2020). External Validation and Transfer Learning of Convolutional Neural Networks for Computed Tomography Dental Artifact Classification. Phys Med Biol.

[52] Chen S, Wang L, Li G, Wu TH, Diachina S, Tejera B (2020). Machine Learning in Orthodontics:Introducing a 3D Auto-segmentation and Auto-landmark Finder of CBCT Images to Assess Maxillary Constriction in Unilateral Impacted Canine Patients. Angle Orthod.

[53] Banumathi A, Raju S, Abhaikumar V (2011). Diagnosis of Dental Deformities in Cephalometry Images Using Support Vector Machine. J Med Syst.

[54] Chen H, Zhang K, Lyu P, Li H, Zhang L, Wu J (2019). A Deep Learning Approach to Automatic Teeth Detection and Numbering Based on Object Detection in Dental Periapical Films. Sci Rep.

[55] Kök H, Acilar AM, İzgi MS (2019). Usage and Comparison of Artificial Intelligence Algorithms for Determination of Growth and Development by Cervical Vertebrae Stages in Orthodontics. Prog Orthod.

[56] Tuzoff DV, Tuzova LN, Bornstein MM, Krasnov AS, Kharchenko MA, Nikolenko SI (2019). Tooth Detection and Numbering in Panoramic Radiographs Using Convolutional Neural Networks. Dentomaxillofac Radiol.

[57] Kuwada C, Ariji Y, Fukuda M, Kise Y, Fujita H, Katsumata A (2020). Deep Learning Systems for Detecting and Classifying the Presence of Impacted Supernumerary Teeth in the Maxillary Incisor Region on Panoramic Radiographs. Oral Surg Oral Med Oral Pathol Oral Radiol.

[58] Sidey-Gibbons JA, Sidey-Gibbons CJ (2019). Machine Learning in Medicine:A Practical Introduction. BMC Med Res Methodol.

[59] Radakovich N, Nagy M, Nazha A (2020). Machine Learning in Haematological Malignancies. Lancet Haematol.

[60] Kotsiantis SB, Zaharakis I, Pintelas P (2007). Supervised Machine Learning:A Review of Classification Techniques. Emerg Artif Intel Appl Comput Eng.

[61] Patcas R, Timofte R, Volokitin A, Agustsson E, Eliades T, Eichenberger M (2019). Facial Attractiveness of Cleft Patients:A Direct Comparison Between Artificial-intelligence-based Scoring and Conventional Rater Groups. Eur J Orthod.

[62] Hwang JJ, Jung YH, Cho BH, Heo MS (2019). An Overview of Deep Learning in the Field of Dentistry. Imaging Sci Dent.

[63] Kebschull M, Guarnieri P, Demmer RT, Boulesteix AL, Pavlidis P, Papapanou PN (2013). Molecular Differences Between Chronic and Aggressive Periodontitis. J Dent Res.

[64] Ozden FO, Ozgonenel O, Ozden B, Aydogdu A (2015). Diagnosis of Periodontal Diseases Using Different Classification Algorithms:A Preliminary Study. Niger J Clin Pract.

[65] Feres M, Louzoun Y, Haber S, Faveri M, Figueiredo LC, Levin L (2018). Support Vector Machine-based Differentiation Between Aggressive and Chronic Periodontitis Using Microbial Profiles. Int Dent J.

[66] Chang HJ, Lee SJ, Yong TH, Shin NY, Jang BG, Kim JE (2020). Deep Learning Hybrid Method to Automatically Diagnose Periodontal Bone Loss and Stage Periodontitis. Sci Rep.

[67] Shimpi N, McRoy S, Zhao H, Wu M, Acharya A (2020). Development of a Periodontitis Risk Assessment Model for Primary Care Providers in an Interdisciplinary Setting. Technol Health Care.

[68] Breslow LA, Aha DW (1997). Simplifying Decision Trees:A Survey. Know Eng Rev.

[69] Elomaa T (1999). The Biases of decision tree pruning strategies. International Symposium on Intelligent Data Analysis.

[70] Punitha S, Al-Turjman F, Stephan T (2021). An Automated Breast Cancer Diagnosis Using Feature Selection and Parameter Optimization in ANN. Comput Electr Eng.

[71] Kwon O, Yong TH, Kang SR, Kim JE, Huh KH, Heo MS (2020). Automatic Diagnosis for Cysts and Tumors of Both Jaws on Panoramic Radiographs Using a Deep Convolution Neural Network. Dentomaxillofac Radiol.

[72] Kononenko I (1994). Estimating attributes:Analysis and extensions of RELIEF. In:European Conference on Machine Learning.

[73] Neville BW, Damm DD, Allen CM, Chi AC (2018). Color Atlas of Oral and Maxillofacial Diseases-E-Book.

[74] Kats L, Vered M, Blumer S, Kats E (2020). Neural Network Detection and Segmentation of Mental Foramen in Panoramic Imaging. J Clin Pediatr Dent.

[75] Lee JH, Han SS, Kim YH, Lee C, Kim I (2020). Application of a Fully Deep Convolutional Neural Network to the Automation of Tooth Segmentation on Panoramic Radiographs. Oral Surg Oral Med Oral Pathol Oral Radiol.

[76] Welch ML, McIntosh C, Purdie TG, Wee L, Traverso A, Dekker A (2020). Automatic Classification of Dental Artifact Status for Efficient Image Veracity Checks:Effects of Image Resolution and Convolutional Neural Network Depth. Phys Med Biol.

[77] Kuwana R, Ariji Y, Fukuda M, Kise Y, Nozawa M, Kuwada C (2021). Performance of Deep Learning Object Detection Technology in the Detection and Diagnosis of Maxillary Sinus Lesions on Panoramic Radiographs. Dentomaxillofac Radiol.

[78] Stehrer R, Hingsammer L, Staudigl C, Hunger S, Malek M, Jacob M (2019). Machine Learning Based Prediction of Perioperative Blood Loss in Orthognathic Surgery. J Craniomaxillofac Surg.

[79] Choi HI, Jung SK, Baek SH, Lim WH, Ahn SJ, Yang IH (2019). Artificial Intelligent Model with Neural Network Machine Learning for the Diagnosis of Orthognathic Surgery. J Craniomaxillofac Surg.

[80] Miki Y, Muramatsu C, Hayashi T, Zhou X, Hara T, Katsumata A (2017). Classification of Teeth in Cone-beam CT Using Deep Convolutional Neural Network. Comput Biol Med.

[81] Štepanovský M, Ibrová A, Buk Z, Velemínská J (2017). Novel Age Estimation Model Based on Development of Permanent Teeth Compared with Classical Approach and Other Modern Data Mining Methods. Forensic Sci Int.

[82] De Tobel J, Radesh P, Vandermeulen D, Thevissen PW (2017). An Automated Technique to Stage Lower Third Molar Development on Panoramic Radiographs for Age Estimation:A Pilot Study. J Forensic Odontostomatol.

[83] Fan F, Ke W, Wu W, Tian X, Lyu T, Liu Y (2020). Automatic Human Identification from Panoramic Dental Radiographs Using the Convolutional Neural Network. Forensic Sci Int.

[84] Niño-Sandoval TC, Pérez SV, González FA, Jaque RA, Infante-Contreras C (2017). Use of Automated Learning Techniques for Predicting Mandibular Morphology in Skeletal Class I, II and III. Forensic Sci Int.

[85] Saghiri MA, Asgar K, Boukani KK, Lotfi M, Aghili H, Delvarani A (2012). A New Approach for Locating the Minor Apical Foramen Using an Artificial Neural Network. Int Endod J.

[86] Saghiri MA, Garcia-Godoy F, Gutmann JL, Lotfi M, Asgar K (2012). The Reliability of Artificial Neural Network in Locating Minor Apical Foramen:A Cadaver Study. J Endod.

[87] Kositbowornchai S, Plermkamon S, Tangkosol T (2013). Performance of an Artificial Neural Network for Vertical Root Fracture Detection:An Ex Vivo Study. Dent Traumatol.

[88] Hiraiwa T, Ariji Y, Fukuda M, Kise Y, Nakata K, Katsumata A (2019). A Deep-learning Artificial Intelligence System for Assessment of Root Morphology of the Mandibular First Molar on Panoramic Radiography. Dentomaxillofac Radiol.

[89] Fukuda M, Inamoto K, Shibata N, Ariji Y, Yanashita Y, Kutsuna S (2019). Evaluation of an Artificial Intelligence System for Detecting Vertical Root Fracture on Panoramic Radiography. Oral Radiol.

[90] Schwendicke F, Rossi JG, Göstemeyer G, Elhennawy K, Cantu AG, Gaudin R (2020). Cost-effectiveness of Artificial Intelligence for Proximal Caries Detection. J Dent Res.

[91] Drummond AM, Ferreira EF, Gomes VE, Marcenes W (2015). Inequality of Experience of Dental Caries Between Different Ethnic Groups of Brazilians Aged 15 to 19 Years. PLoS One.

[92] Shackleton N, Broadbent JM, Thornley S, Milne BJ, Crengle S, Exeter DJ (2018). Inequalities in Dental Caries Experience Among 4-year-old New Zealand Children. Community Dent Oral Epidemiol.

[93] Nogueira JS, Pereira AC, Frias AC, Ambrosano GM, Cortellazzi KL, Guerra LM (2019). Social Capital and Factors Associated with the Caries Experience in Adults-a Population-Based Study in Brazil. Braz Oral Res.

[94] Cheng C, Cheng X, Dai N, Jiang X, Sun Y, Li W (2015). Prediction of Facial Deformation After Complete Denture Prosthesis Using BP Neural Network. Comput Biol Med.

[95] Raith S, Vogel EP, Anees N, Keul C, Güth JF, Edelhoff D (2017). Artificial Neural Networks as a Powerful Numerical Tool to Classify Specific Features of a Tooth Based on 3D Scan Data. Comput Biol Med.

[96] Wei J, Peng M, Li Q, Wang Y (2018). Evaluation of a Novel Computer Color Matching System Based on the Improved Back-propagation Neural Network Model. J Prosthodont.

[97] Abdalla-Aslan R, Yeshua T, Kabla D, Leichter I, Nadler C (2020). An Artificial Intelligence System Using Machine-learning for Automatic Detection and Classification of Dental Restorations in Panoramic Radiography. Oral Surg Oral Med Oral Pathol Oral Radiol.

[98] Yamaguchi S, Lee C, Karaer O, Ban S, Mine A, Imazato S (2019). Predicting the Debonding of CAD/CAM Composite Resin Crowns with AI. J Dent Res.

[99] Cui Q, Chen Q, Liu P, Liu D, Wen Z (2020). Clinical Decision Support Model for Tooth Extraction Therapy Derived from Electronic Dental Records. J Prosthet Dent.

[100] Hadj Saïd M, Le Roux MK, Catherine JH, Lan R (2020). Development of an Artificial Intelligence Model to Identify a Dental Implant from a Radiograph. Int J Oral Maxillofac Implants.

[101] Papantonopoulos G, Gogos C, Housos E, Bountis T, Loos BG (2017). Prediction of Individual Implant Bone Levels and the Existence of Implant “Phenotypes”. Clin Oral Implants Res.

[102] Schwendicke F, Krois J (2021). Better Reporting of Studies on Artificial Intelligence:CONSORT-AI and Beyond. J Dent Res.

[103] Bellazzi R, Ferrazzi F, Sacchi L (2011). Predictive Data Mining in Clinical Medicine:A Focus on Selected Methods and Applications. Wiley Interdiscip Rev Data Min Know Discov.

[104] Blum AL, Langley P (1997). Selection of Relevant Features and Examples in Machine Learning. Artif Intel.

[105] Bloedorn E, Michalsi RS (1998). Data-driven Constructive Induction. IEEE Intel Sys Appl.

[106] Park SH, Han K (2018). Methodologic Guide for Evaluating Clinical Performance and Effect of Artificial Intelligence Technology for Medical Diagnosis and Prediction. Radiology.

[107] Vayena E, Blasimme A, Cohen IG (2018). Machine Learning in Medicine:Addressing Ethical Challenges. PLoS Med.

[108] Cabitza F, Rasoini R, Gensini GF (2017). Unintended Consequences of Machine Learning in Medicine. JAMA.

[109] Moons KG, Altman DG, Reitsma JB, Ioannidis JP, Macaskill P, Steyerberg EW (2015). Transparent Reporting of a Multivariable Prediction Model for Individual Prognosis or Diagnosis (TRIPOD):Explanation and Elaboration. Ann Intern Med.

[110] Chawla NV, Bowyer KW, Hall LO, Kegelmeyer WP (2002). SMOTE:Synthetic Minority Over-sampling Technique. J Artif Intell Res.

[111] López V, Fernández A, Moreno-Torres JG, Herrera F (2012). Analysis of Preprocessing vs. Cost-sensitive Learning for Imbalanced Classification. Open Problems on Intrinsic Data Characteristics. Expert Syst Appl.

[112] Huang C, Li Y, Loy CC, Tang X (2016). Learning deep representation for imbalanced classification. In:Proceedings of the IEEE Conference on Computer Vision and Pattern Recognition.

[113] Bossuyt PM, Reitsma JB, Bruns DE, Gatsonis CA, Glasziou PP, Irwig L (2015). STARD, 2015:An Updated List of Essential Items for Reporting Diagnostic Accuracy Studies. Clin Chem.

[114] Walsh T (2018). Fuzzy Gold Standards:Approaches to Handling an Imperfect Reference Standard. J Dent.

[115] Chikere CM, Wilson K, Graziadio S, Vale L, Allen AJ (2019). Diagnostic Test Evaluation Methodology:A Systematic Review of Methods Employed to Evaluate Diagnostic Tests in the Absence of Gold Standard-an Update. PLoS One.

[116] Pethani F (2020). Promises and Perils of Artificial Intelligence in Dentistry. Aust Dent J.

[117] Wiens J, Saria S, Sendak M, Ghassemi M, Liu VX, Doshi-Velez F (2019). Do No Harm:A Roadmap for Responsible Machine Learning for Health Care. Nat Med.

[118] He J, Baxter SL, Xu J, Xu J, Zhou X, Zhang K (2019). The Practical Implementation of Artificial Intelligence Technologies in Medicine. Nat Med.

